# Effect of Virtual Reality Hypnosis on Pain Threshold and Neurophysiological and Autonomic Biomarkers in Healthy Volunteers: Prospective Randomized Crossover Study

**DOI:** 10.2196/33255

**Published:** 2022-07-29

**Authors:** Claire Terzulli, Meggane Melchior, Laurent Goffin, Sylvain Faisan, Coralie Gianesini, Denis Graff, André Dufour, Edouard Laroche, Chloé Chauvin, Pierrick Poisbeau

**Affiliations:** 1 HypnoVR Strasbourg France; 2 Institut des Neurosciences Cellulaires et Intégratives Centre National de la Recherche Scientifique University of Strasbourg Strasbourg France; 3 ICube Laboratory University of Strasbourg Strasbourg France; 4 Anesthesiology Clinique Rhéna Strasbourg France; 5 Laboratoire de Neurosciences Cognitives et Adaptatives Centre National de la Recherche Scientifique University of Strasbourg Strasbourg France; 6 Department of Anesthesiology and Intensive Care University Hospital of Strasbourg Strasbourg France

**Keywords:** virtual reality, hypnosis, pain, analgesia, autonomic changes, thermal pain, physiological, nervous system, heat pain

## Abstract

**Background:**

Virtual reality hypnosis (VRH) is a promising tool to reduce pain. However, the benefits of VRH on pain perception and on the physiological expression of pain require further investigation.

**Objective:**

In this study, we characterized the effects of VRH on the heat pain threshold among adult healthy volunteers while monitoring several physiological and autonomic functions.

**Methods:**

Sixty healthy volunteers were prospectively included to receive nociceptive stimulations. The first set of thermal stimuli consisted of 20 stimulations at 60°C (duration 500 milliseconds) to trigger contact heat evoked potentials (CHEPs). The second set of thermal stimuli consisted of ramps (1°C/second) to determine the heat pain threshold of the participants. Electrocardiogram, skin conductance responses, respiration rate, as well as the analgesia nociception index were also recorded throughout the experiment.

**Results:**

Data from 58 participants were analyzed. There was a small but significant increase in pain threshold in VRH (50.19°C, SD 1.98°C) compared to that in the control condition (mean 49.45°C, SD 1.87; *P*<.001, Wilcoxon matched-pairs signed-rank test; Cohen *d*=0.38). No significant effect of VRH on CHEPs and heart rate variability parameters was observed (all *P*>0.5; n=22 and n=52, respectively). During VRH, participants exhibited a clear reduction in their autonomic sympathetic tone, as shown by the lower number of nonspecific skin conductance peak responses (*P*<.001, two-way analysis of variance; n=39) and by an increase in the analgesia nociception index (*P*<.001, paired *t*-test; n=40).

**Conclusions:**

The results obtained in this study support the idea that VRH administration is effective at increasing heat pain thresholds and impacts autonomic functions among healthy volunteers. As a nonpharmacological intervention, VRH has beneficial action on acute experimental heat pain. This beneficial action will need to be evaluated for the treatment of other types of pain, including chronic pain.

## Introduction

Pain is an unpleasant sensory and emotional experience that is essential to the survival of living beings; however, its usefulness is lost if pain becomes chronic (duration>3 months) [1]. For this reason, chronic pain is classified as a disease by the World Health Organization (under the International Classification of Diseases 11th edition) [2]. Chronic pain is a heavy burden as it is associated with several comorbidities, ranging from emotional disturbances to severe psychosocial disorders. Furthermore, pharmacological treatments of chronic pain are often unsatisfactory, which reinforces the recommendations from pain societies across the world to suggest nonpharmacological interventions as an adjunct modality for pain management. Several of these interventions have already proven to have some efficacy in addressing various pain states, including (but not restricted to) modulation of attention, hypnosis, musicotherapy, or physical exercises [3-6]. The development of digital tools such as virtual reality (VR) portable systems represents a unique opportunity for the treatment of pain, as these tools can combine several of these nonpharmacological treatments/methods in easy-to-use eHealth solutions.

Fully immersive VR headsets isolate users from the “real world” and move them into an enjoyable alternative 3D virtual world. If well-executed, VR environments have the capability to reduce pain, as demonstrated by the pioneering work from Hoffman and collaborators [7] and recently reviewed by Chuan et al [8]. The specific mechanisms underlying this analgesic action are not fully understood, but are likely to involve several neural functions, and in particular pathways originating from the opioidergic-sensitive frontal cortex area, modulating attentional processes [6]. These projections innervate several subcortical structures known to shape the emotional responses (eg, the amygdala) and recruit the descending inhibitory control to limit sensorispinal nociceptive integration. In line with this mechanism, distraction-oriented tasks lead to activation of the amygdala that is directly correlated with a reduction of pain scores [9]. One of the rare imaging studies available [10] suggested that the activation of pain-processing structures (anterior cingulate cortex, somatosensory cortex S1, insula, thalamus) is significantly reduced by VR, which likely explains the observed reduction in pain scores after experimental noxious heat stimulations [10]. Based on these results and structures with reduced activity, it can be hypothesized that several components (ie, sensory-discriminative, affective-emotional, and cognitive) are modulated by VR. It is likely that other pathways and analgesic mechanisms remain to be discovered to explain the observed effects on pain responses.

Apart from modulation of the cortical nociceptive processing giving rise to the sensation of pain and its emotional value, pain motor responses may also be modulated in their somatic (conscious) or autonomic components. Because autonomic motor responses are less sensitive to subjective cues, they are often used in combination with other evaluation pain scales relying on subject impressions. The underlying mechanisms of VR action seem to be even more complex with the latest VR devices that often combine distracting visual cues with analgesia-promoting auditory sensory stimulations, ranging from passive listening of music to hypnotic suggestions [3,4,11]. Altogether, VR is likely to be an interesting tool to reduce pain and its unpleasantness.

In this study, we used virtual reality hypnosis (VRH), which combines the computer-generated immersive environment of VR with a hypnotic script [12]. Similar to classical hypnosis treatments, the VRH scheme used in this study consists of an “induction period” (usually an invitation to focus one’s attention) and a “dissociation” period (separation between the mental and the environment), followed by suggestions of pain reduction as analgesia. Although the beneficial effect of hypnosis on pain has been previously demonstrated [8,13], only a few studies have investigated VRH effects on pain levels [14-17]. Here, we used nociceptive heat stimulations triggered by an ultrafast thermode (TCS-II, QST-Lab; 300°C/second) to evaluate the effects of VRH (HypnoVR) on pain threshold and on contact heat evoked potentials (CHEPs).

Our hypothesis was that this VRH device, combining VR and hypnotic suggestions, increases pain thresholds, an effect that can possibly be predicted by cortical electrophysiological signatures and autonomic monitoring. Thus, the secondary objectives consisted of analyzing VRH-associated changes of several physiological biomarkers such as heart rate and heart rate variability, analgesia nociception index, respiratory rate, and skin conductance responses (SCRs) with and without VRH.

## Methods

### Participants

Sixty adult participants were included in the study (32 women and 28 men). Participants had to be affiliated with the French social security system, and could not participate if they had unbalanced epilepsy, psychotic disorders, depression, hearing and/or visual impairments preventing the use of VRH, or chronic diseases that may influence pain perception (eg, chronic pain, diabetes); if they were participating in another clinical study; were unable to provide informed consent; or refused to participate. Women could not participate if they were pregnant or breastfeeding.

### Ethics Considerations

This study was approved by the ethics review board of CPP OUEST IV-Nantes (approval date March 28, 2019; Agence Nationale de Sécurité du Médicament et des Produits de Santé, French Ministry of Health, information date May 7, 2019; IdRCB n° 2018-A02992-53). All participants signed a written informed consent form prior to participation.

### Study Design

This was an open, single-center, comparative, crossover study. Each participant performed the experiment in both the VRH and control (without VRH) conditions. To limit a potential order effect, the time of VRH application was randomly counterbalanced across participants. At the time of inclusion, suggestibility was assessed using the standardized Barber scale test [18] and the anxiety trait was assessed using the State-Trait Anxiety Inventory (STAI) self-administered questionnaire [19]. During the experiment, participants were exposed to the different thermal stimulation protocols during the control or VRH condition. Cardiac frequency, respiration rate, electrodermal conduction, and analgesia nociception index (ANI; MDoloris, France) were recorded throughout all experiments. Occurrence of adverse events was also recorded.

### VRH Setup

For the experiment, the VR headset was an Oculus Rift (resolution: 1080×1200 pixels per eye; field of view: 110°; frame rate: 90 Hz) coupled to a laptop computer (Asus GL502VS managed by an IntelCore i7-6700HQ processor at 2.6 GHz; RAM: 16 GB; graphics card: Nvidia GeForce GTX 1070; Windows 10 64-bit). The sound was delivered by the Oculus Rift headset.

VRH was delivered through the HypnoVR application, coupling 3D immersive and dynamic visual scenery (walking on a beach or scuba diving) with a standardized prerecorded hypnotic script (including relaxing and analgesic suggestions available in several languages) as well as musical background following music therapy principles. The hypnotic script was the same for all environmental scenarios. The musical piece followed a U-shape sequence so that, together with visual experiences, it progressively helped the participants reach a state of cardiac coherence [20]. Participants were given the opportunity to choose between one of the two preferred visual sceneries, a male or female voice for hypnotic suggestions, and musical background among three melodies. The VRH session lasted 20 minutes. The first 3 minutes correspond to the “induction period,” a sequence aiming to focus an individual’s attention with deep-breathing exercises. This was followed by well-being and pain relief suggestions (including changing pain sensations into something else, reduction in pain, optimization of well-being, changes in focus attention away from pain, and increased ability to ignore pain). The session ended with a 2-minute sequence leading the participant back to a normal conscious state.

### Thermal Stimuli and Experimental Protocol

**Figure 1:**
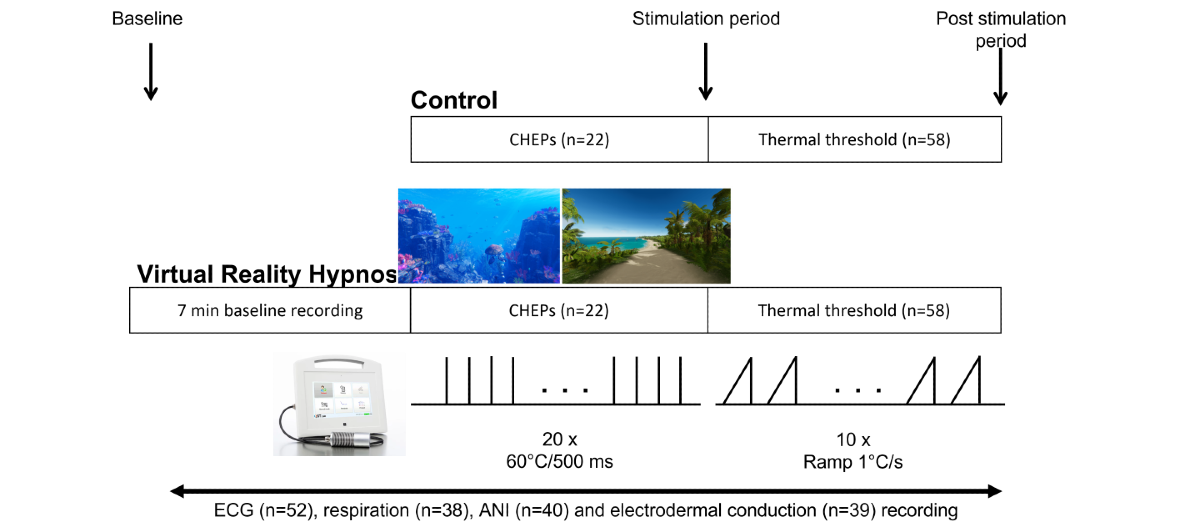
Overview of the experimental protocol indicating 3 periods: virtual reality hypnosis induction, stimulation protocols with acute heat stimulation (for somatosensory event-related potential measures), and temperature ramps (for pain threshold determination). Arrows indicate the period of measurements of autonomic parameters. Two representative images of the virtual environment proposed to patients are shown. n corresponds to the number of subjects included in each analysis. ANI: analgesia nociception index; CHEP: contact heat evoked potential; ECG: electrocardiogram.

Thermal hot stimuli were applied with a thermal stimulator (TCS II, QST.Lab, Strasbourg, France) following the general scheme indicated in Figure 1. The thermode was placed on the dorsal surface of the nondominant hand. The first stimulation session was used to evaluate CHEPs, comprising 20 transient stimulations at 60°C/500 milliseconds. The temperature increase rate was set at 300°C/second and the decrease rate was set at 200°C/second. The baseline temperature was set at 30°C. To avoid increased sensibility of the stimulated area, the thermode was moved from one contact field stimulation to another (always on the dorsal part of the nondominant hand).

This session was followed by a second stimulation session used to evaluate heat pain thresholds, which was performed using the limit method with 10 ascending ramps. The temperature was increased at a rate of 1°C/second, from skin temperature (measured before the first ramp) to a temperature that volunteers considered as painful. Volunteers were given the instruction to stop the temperature increments with a push button when they felt that the stimulation was becoming painful. The pain threshold was assessed by averaging 10 trials. To take into account interindividual differences in skin temperature, the absolute pain threshold was measured as well as the difference between the skin temperature (measured just before the first ramp) and the absolute value of the threshold (Δ temperature). All participants were subjected to the hot stimulation sequence, enabling measurements of both CHEPs and pain thresholds with and without VRH. Stimuli occurred after a few minutes (maximum 7 minutes) of rest.

### Physiological Data

Cortical CHEPs (from electroencephalography [EEG] data) were recorded using Active Two AD-Box coupled with a 32–active electrodes cap respecting the 10/20 system (Biosemi). Ground electrodes (common mode sense and a driven right leg) were located between C3-Cz and between Cz-C4, respectively. The sampling frequency was set to 2048 Hz. EEG data were collected and monitored throughout the recording with Actiview version 8.0 (BioSemi B.V., WG-Plein 129, 1054SC). Raw data were preprocessed (offline) with Cartool software [21] with the help of a 50-Hz notch filter combined to 0.1-Hz high- and 80-Hz low-pass filters. The EEG file was then segmented into discrete single-trial epochs, 1500 milliseconds long with a 500-millisecond baseline before stimulus onset and a 1000-millisecond poststimulus period. Successful trials were averaged for each participant. Movement and eye-blink artifacts were removed manually. Data were checked individually for flat or noisy periods with Cartool software before further analysis.

Physiological parameters were recorded with BIOPAC MP150 (BIOPAC System Inc). The electrocardiogram (ECG; beats/minute) was measured with BIOPAC ECG100C. The breathing rate (cycles/minute) was measured by a thermistor that determined the difference in temperature between inhaled and exhaled air (BIOPAC TSD202F). SCRs were measured from the extremities of the index and middle fingers of the dominant hand (BIOPAC TSD203). Acquisition was performed through a homemade software collecting and synchronizing data from the BIOSEMI and BIOPAC acquisition equipment. The sampling rate was set to 500 Hz for BIOPAC signals. Preprocessing of ECG included a 5th-order Butterworth high-pass filter of 0.5 Hz and a 50-Hz Notch filter. The detection of R-R peak intervals enabled extracting the percentage of successive R-R intervals that differ by more than 50 milliseconds (pNN50) and the root mean square of successive R-R interval differences (RMSSD) was calculated. A 3rd-order finite impulse response filter was applied to remove the electrical noise for SCR. A simple pic detection was performed for respiration data with no additional data treatment. ANI scores were calculated by the mDoloris monitor (MetroDoloris) following a previously reported method [22].

ECG, respiration, and SCR (all synchronized within one file) were epoched in a 1-minute-long file. Epoched data were analyzed with Clampfit (Molecular Devices) and Python 3.8 (especially the Neurokit library [23]).

### Data Collection

Data were prospectively collected using an audit form established for the study. All personal identifying information was removed from the database in accordance with regulations prescribed by the French data protection authority Commission Nationale de l’Informatique et des Libertés (CNIL 2213128). Collected data included the demographic characteristics (age, sex, education level) and if the participants had previously experienced motion sickness, as it might be a risk factor for nausea during VRH.

### Statistical Analysis

Results are expressed as mean (SD). The statistical analyses included a descriptive component and an analytical component. All statistical analyses were performed with GraphPad Prism software (version 6). The significance level was set at α=.05 for all analyses. Normality of the distributions was tested using the Shapiro-Wilk normality test. Differences between male and female participants in baseline characteristics were analyzed using the Mann-Whitney *U* test, unpaired *t*-test, or Fisher exact test, as applicable. Cohen *d*, which specifically measures the effect size of the difference between two means, was calculated as (mean 1–mean 2)/pooled SD for both groups; thresholds of 0.3, 0.5, and 0.8 were considered as a small, medium, and large effect size, respectively. Differences in thermal sensitivity between the control and VRH conditions were assessed using the Wilcoxon matched-pairs signed-rank test. CHEPs were analyzed with a Student *t*-test for paired data and with two-way analysis of variance (ANOVA) followed by the Tukey posthoc test. Heart rate, respiration rate, and skin conductance data were analyzed with two-way ANOVA followed by posthoc Dunn-Sidak multiple-comparison correction. Statistical significance of the heart rate variability parameters, pNN50, and RMSSD was assessed using a Wilcoxon matched-pairs signed-rank test. ANI was analyzed with a paired *t*-test. To assess the effect of VRH, results were compared with the control condition. Note that the number of analyzed participants varies for different analyses because of unexpected and random electrical artifacts appearing in the periods of interest (ie, around the trigger time). If such artifacts occurred, we only retained uncontaminated signals to perform the analysis for a given participant.

## Results

### Participant Characteristics

**Figure 2:**
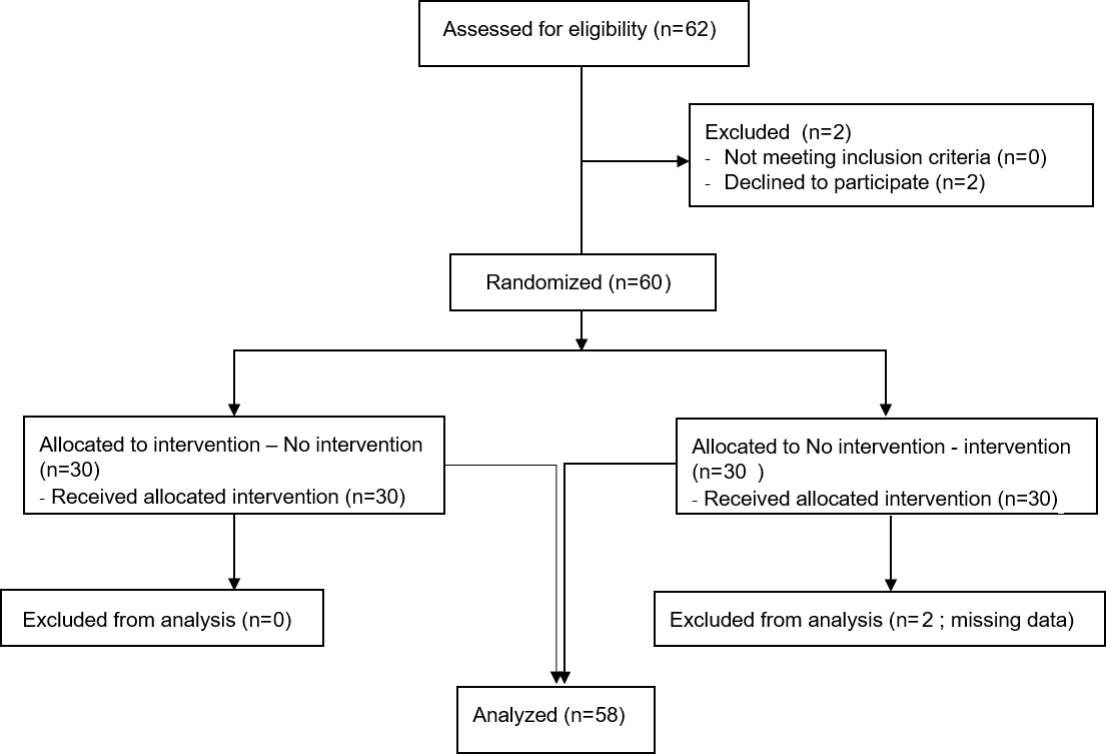
Flow diagram of screened, randomized, and excluded participants.

A total of 60 participants fulfilled the inclusion criteria and were included in the study. Two participants (1 man and 1 woman) were excluded owing to incomplete data. Thus, 58 participants were included in the final analysis (Figure 2), including 27 men and 31 women. The mean age was 30 years, which ranged from 19 to 56 years (Table 1). There were no significant age or sex correlations with pain threshold measures, CHEPs, or autonomic parameters.

Most participants (40/58, 69%) did not previously suffer from travel sickness. The Barber suggestibility scores ranged from 0 to 7 out of a possible total of 8, with a mean of 3.2. Men and women had similar Barber suggestibility scores, demographic characteristics, and baseline measured variables, except for the STAI score and history of motion sickness, which were slightly higher in women. No adverse events were reported by any subject during the study.

**Table 1:** Participant characteristics.

Characteristics	All participants (N=58)	Range (Total)	Women (n=31)	Men (n=27)	*P* value (women vs men)
Age (years), mean (SD)	30 (9.4)	19-56	31 (10.4)	29 (8.1)	.66^a^
Education (years postbac^b^), mean (SD)	4.3 (2.3)	1-8	3.8 (2.6)	4.8 (1.9)	.08^a^
STAI^c^ (score/80), mean (SD)	37.8 (9.2)	23-60	39.9 (8.6)	35.3 (9.4)	.03^a^
Barber (score/8), mean (SD)	3.2 (1.6)	0-7	3.3 (1.6)	3.1 (1.6)	.65^d^
Travel sickness history, n	18	N/A^e^	15	3	.004^f^
BMI, mean (SD)	22.5 (7.0)	18 – 32.4	21.9 (2.7)	23.2 (3.3)	.13^a^

^a^Mann-Whitney *U* test.

^b^After undergraduate college degree.

^c^STAI: State-Trait Anxiety Inventory.

^d^Unpaired *t*-test.

^e^N/A: not applicable.

^f^Fisher exact test.

### VRH Effect on Pain Thresholds and Pain Evoked Potentials

**Figure 3:**
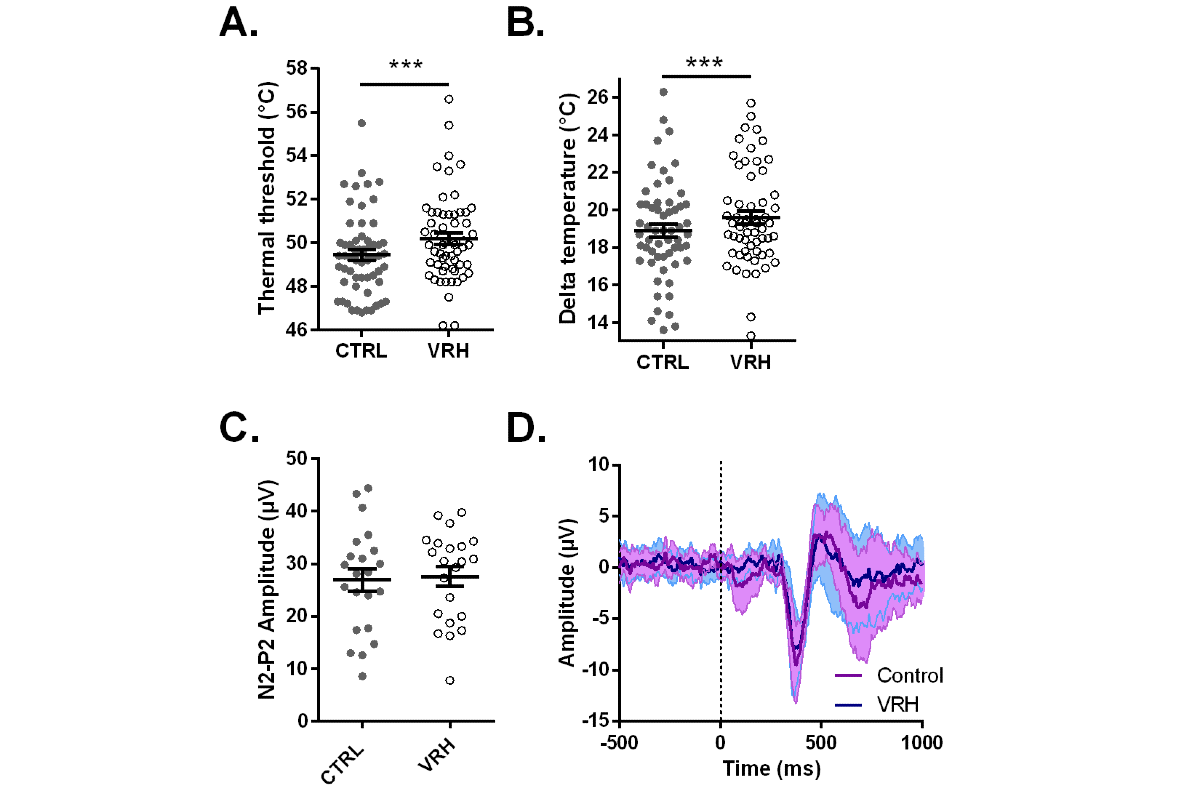
Effect of virtual reality hypnosis (VRH) on pain thresholds and somatosensory event-related potentials. A. Mean (SEM) absolute temperature before and after VRH. B. Mean (SEM) delta temperature (ie, the difference between the temperature threshold and skin temperature) in both conditions. C. Evolution of the mean (SEM) amplitude of N2-P2 (in µV) between control and VRH conditions. D. Superimposed mean traces of somatosensory event-related potentials obtained during VRH and without VRH, represented with their respective SDs. ***P<.001; Wilcoxon matched-pairs rank test (N=58). CTRL: control group, without VRH.

As illustrated in Figure 3A and B, we observed significantly higher mean temperature thresholds (49.45°C, SD 1.87 in the control and 50.19°C, SD 1.98 in the VRH group; Wilcoxon matched-pairs signed-rank test *P*<.001; N=58) and Δ temperature (18.91°C, SD 2.65 in the control and 19.59°C, SD 2.58 in the VRH group; Wilcoxon matched-pairs signed-rank test *P*<.001; N=58) in the VRH compared to control condition. The mean absolute difference between the control and VRH conditions was 0.74°C (95% CI 0.43-1.06), and the mean difference in Δ temperatures was 0.68°C (95% CI 0.28-1.08). Cohen *d* calculation yielded a small effect size of 0.384.

We then measured CHEPs during the control and VRH conditions. Mean epoch traces for all participants with more than two successful trials in both conditions were retained in the analysis and are shown in Figure 3D (mean 3.5, SD 1.8 successful trials; range 2-8). The mean N2-P2 amplitude was 26.9 (SD 9.92) µV and 27.6 (SD 8.66) µV in the control and VRH condition, respectively (Figure 3C). No significant difference in the N2-P2 amplitude between the two conditions was found (mean of differences 0.67, 95% CI –1.59 to 2.94; t_21_=0.62, *P*=.54; n=22).

### VRH Modulation of Pain-Induced Physiological Changes

**Figure 4:**
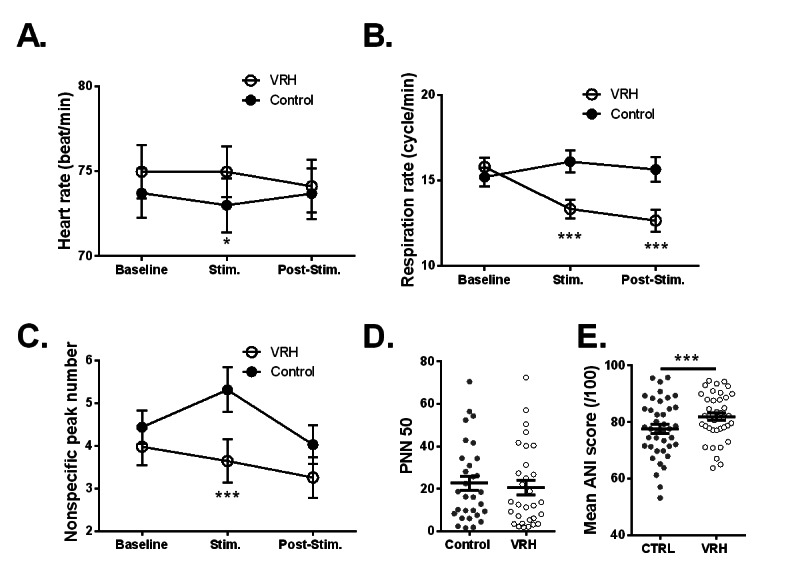
Effect of virtual reality hypnosis (VRH) on autonomic parameters (mean, SEM). Mean heart rate (A), respiration rate (B), and nonspecific skin peak conductance (C) at baseline, during stimulation (Stim.; ie, between somatosensory event-related potential stimulations and ramps), and after the last stimulation (Post-Stim.) for the control and VRH conditions. D. Percentage of successive R-R intervals that differ by more than 50 milliseconds (PNN50) as an index of cardiac variability. E. Analgesia nociception index (ANI) for both conditions. *P<.10, ***P<.001 with Sidak multiple comparison test for panels A to C; ***P<.001 with paired t-test for panel E.

Mean values for heart rate, respiration rate, nonspecific SCR, heart rate variability parameters (ie, pNN50, RMSSD, and ANI) are shown in Figure 4 in various conditions.

The mean heart rate remained globally stable during the entire protocol, which was divided into a baseline period, a period corresponding to the intervals between the stimulation protocols, and a period occurring after the stimulation protocols. No differences were observed between the control and VRH conditions for each period (Figure 4A; two-way ANOVA time×condition *F*_2, 102_=1.31, *P*=.27; n=52). The respiration rate was similar at baseline for both conditions. No change in the respiratory rate was observed during the three phases of the analysis for the control group, whereas a significant decrease in this rate was observed in the VRH condition during the stimulation period and shortly after, at the end of the recording period (Figure 4B; two-way ANOVA time×condition *F*_2,74_=13.71, *P*<.001; n=38). As for the other parameters, SCR was similar at baseline for both conditions, although the number of nonspecific peaks increased during the stimulation period in the control condition. This increase was not observed in the VRH condition and mean values remained stable (Figure 4C; two-way ANOVA condition *F*_1,38_=13.74, *P*<.001; n=39). The mean value for the heart rate variability parameter pNN50 remained similar in the VRH and control conditions (Figure 4D; Wilcoxon matched-pairs signed-rank test *P*=.12; n=40). No change in RMSSD was observed (Wilcoxon test *P*=.96, n=40; control mean 36.81, SD 24.32; VRH mean 37.25, SD 20.56). This was not the case for ANI mean values, which significantly increased in the VRH condition compared to the control (Figure 4F; paired *t*-test t_39_=3.76, *P*<.001; n=40). Interestingly, the ANI score is calculated by using the ratio between low-frequency parasympathetic and high-frequency sympathetic frequency powers. This parameter has been shown to be more sensitive in pain states and discomfort [20].

## Discussion

This study investigated the effect of VRH on pain thresholds and CHEPs in response to heat stimuli in healthy adult volunteers (men and women). Changes in several physiological parameters were also monitored during the stimulation protocol. We found that VRH increased the heat pain threshold, reduced the mean respiratory rate during the VRH session, and increased the ratio between parasympathetic and sympathetic tones, as seen by the stability of SCR and the increase in ANI score.

Our findings are consistent with a recently published study, in which the authors measured the effect of VR on heat-pain tolerance limits [24]. They tested two VR conditions: (1) an immersive condition in which the viewer could experience a 360° video and audio immersion, and (2) a nonimmersive condition with audio and 2D video only. They found an increase in the pain tolerance threshold in both conditions, which was higher with the immersive VR (by approximately 1°C). The effect size on pain threshold for our study and this previous work of Colloca et al [24] was small and of similar amplitude (Cohen *d* 0.384 and 0.321, respectively). In addition, another study showed a decrease in worst pain intensity and pain unpleasantness following VR in response to thermal stimulation of the foot [25]. Interestingly, the increase in pain threshold reported by healthy volunteers in this study was not associated with changes in CHEPs amplitude. CHEPs result from the activation of different cortical structures, including the somatosensory, insular, and cingulate cortices [26], but are also modulated by attentional processes and stimulus salience [27]. This likely suggests that the cortical processing of the heat stimulus was not modulated by VRH. One possible explanation is that VRH did not sufficiently distract the participant’s attention to affect the CHEPs amplitude. However, VRH might still impact the activity of subcortical structures involved in the modulation of pain and in its perception as a negative emotion [6,28,29]. This working hypothesis is in line with a recent study showing that active VR (eg, a game) but not passive VR (eg, a movie) decreases brain activity following painful electrical stimuli, which was associated with a reduction in the experience of pain [30]. In this study, we did not observe any effect of VRH on pain scores despite a significant difference in heat pain threshold. Associating the measurement of pain sensation with brain imaging could provide further information on the mechanisms underlying the effects of VRH on pain. VR (but not VRH) effects were investigated in one study using functional magnetic resonance imaging brain scans, in which reductions in the activity of key structures of the pain matrix were observed [10].

Taking advantage of the simultaneous recording of some physiological parameters under VRH, we could observe significant changes of certain signals even though the nociceptive stimulations were of short duration. We observed a decrease in respiration rate and in SCR in VRH compared to the control condition, which confirmed the efficiency of the VRH script to promote relaxation and a possible decreased anxiety level that is known to reduce pain [31]. SCR peaks, likely reflecting sympathetic activation and arousal, were also lowered in our study [32]. This effect may also account for a reduction in arousal contributing to the effect observed on pain thresholds [33]. This finding is in line with the elevation of the ANI score, which is used to monitor the comfort (ie, parasympathetic tone). However, we failed to detect any effect in temporal-domain heart rate variability parameters. This lack of effect was also reported in a recent study, where the SD from normal to normal was affected only in one VR condition (immersive Ocean) but not in the others [24].

Compared to other VR devices, the VRH device used in this study includes not only visual and auditory immersive clues but also a hypnotic script following the classical hypnosis sequence for treatment purpose (ie, induction, dissociation, and suggestions of pain reduction). Hypnosis is an active cognitive treatment that allows the mind to influence sensations and perceptions of the body [30]. Accordingly, the interaction between the patient and the therapist aims to engage the patient in cognitive processes to reduce pain. The efficacy of VRH has also been demonstrated in patients suffering from traumatic pain or burn pain [15,34] and a case report suggested a positive effect on neuropathic pain [14].

The main limitation of this study is linked to the experimental setup, as the hardware/software connections generated electrical artifacts in some cases so that the data from several participants could not be properly analyzed and were thus withdrawn from a specific analysis. Another limitation concerns the characteristics of the enrolled participants, who were healthy volunteers, highly educated, and of young age, which is not representative of the general population [35-37]. Finally, we measured responses to heat stimulations applied with a quantitative sensory testing apparatus in a nonstressful laboratory environment, which does not correspond to the clinical reality of pain, regardless of whether it is acute or chronic. For example, procedural pain also implies that the individual is experiencing other forms of stress and discomfort linked to olfactory, auditory, or visual cues induced by the medical procedure. In this context, VRH might be even more beneficial by removing patients from these cues, redirecting their attention to a more pleasant environment with analgesic hypnotic suggestions [3,11]. With regard to chronic pain states, which are often associated with emotional comorbidities, it would be of interest to study the effect of repeated VRH sessions when the device is freely accessible to patients (and not only the effect of a single VRH session as performed in this study). This will enable recording the improvement in subjective pain score (intensity and unpleasantness) over time as well as the impact on quality of life.

Collectively, the results of this study suggest that VRH has a small but significant beneficial effect on acute heat pain. This effect of VRH may involve multiple modulatory pathways, modifying the perception of pain and its expression through conscious and autonomic parameters, all leading to a better relaxation state. Acute or repeated use of VR might hence provide therapeutic benefits in patients suffering from pain, including when they are outside a hospital structure, as found in recent studies [38,39]. This will require further investigations; however, compared to other nonpharmacological interventions, VRH has the advantage of being easy to use and available at home for repeated use (after a short session of therapeutic education) without the need of any medical assistance.

## Abbreviations

ANI: analgesia nociception index
ANR: Agence Nationale de la Recherche (French national research agency)
ANOVA: analysis of variance
CHEP: contact heat evoked potential
ECG: electrocardiogram
EEG: electroencephalogram
pNN50: percentage of successive R-R intervals that differ by more than 50 milliseconds
RMSSD: root mean square of successive R-R interval differences
SCR: skin conductance response
STAI: State-Trait Anxiety Inventory
VR: virtual reality
VRH: virtual reality hypnosis
